# Single-cell sequencing elucidates the mechanism of NUSAP1 in glioma and its diagnostic and prognostic significance

**DOI:** 10.3389/fimmu.2025.1512867

**Published:** 2025-02-05

**Authors:** Meng-Yu Zhao, Zhao-Lei Shen, Hongzhen Dai, Wan-Yan Xu, Li-Na Wang, Yu- Gu, Jie-Hui Zhao, Tian-Hang Yu, Cun-Zhi Wang, Jia-feng Xu, Guan-Jun Chen, Dong-Hui Chen, Wen-Ming Hong, Fang Zhang

**Affiliations:** ^1^ Department of Neurosurgery, First Affiliated Hospital of Anhui Medical University, Hefei, China; ^2^ School of Nursing, Anhui Medical University, Hefei, China; ^3^ Research and Experiment Center of Anhui Medical University, Anhui Medical University, Hefei, China; ^4^ Department of Neurosurgery, Lu’an People’s Hospital, Luan, China; ^5^ Open Project of Key Laboratory of Dermatology, Ministry of Education, Anhui Medical University, Hefei, China

**Keywords:** glioma, NUSAP1, cancer immunology, single-cell sequencing, molecular mechanisms

## Abstract

**Background:**

Personalized precision medicine (PPPM) in cancer immunology and oncology is a rapidly advancing field with significant potential. Gliomas, known for their poor prognosis, rank among the most lethal brain tumors. Despite advancements, there remains a critical need for precise, individualized treatment strategies.

**Methods:**

We conducted a comprehensive analysis of RNA-seq and microarray data from the TCGA and GEO databases, supplemented by single-cell RNA sequencing (scRNA-seq) data from glioma patients. By integrating single-cell sequencing analysis with foundational experiments, we investigated the molecular variations and cellular interactions within neural glioma cell subpopulations during tumor progression.

**Results:**

Our single-cell sequencing analysis revealed distinct gene expression patterns across glioma cell subpopulations. Notably, differentiation trajectory analysis identified NUSAP1 as a key marker for the terminal subpopulation. We found that elevated NUSAP1 expression correlated with poor prognosis, prompting further investigation of its functional role through both cellular and animal studies.

**Conclusions:**

NUSAP1-based risk models hold potential as predictive and therapeutic tools for personalized glioma treatment. In-depth exploration of NUSAP1’s mechanisms in glioblastoma could enhance our understanding of its response to immunotherapy, suggesting that targeting NUSAP1 may offer therapeutic benefits for glioma patients.

## 1 Introduction

Gliomas represent the most common malignant tumors within the central nervous system, accounting for more than 30% of all primary brain tumors, and are characterized by high rates of incidence and mortalit (1). Although the precise cause remains unknown, genetic predispositions, environmental factors, and gene mutations are thought to significantly contribute to glioma development (2, 3). Pathologically, gliomas are highly heterogeneous and aggressive, especially in high-grade forms like glioblastoma (GBM), known for rapid growth and the ability to invade surrounding brain tissue (4). Despite standard treatments such as surgery, radiotherapy, and chemotherapy, the prognosis for most glioma patients is bleak, with a five-year survival rate below 10% (5). Additionally, gliomas’ resistance to conventional therapies highlights the urgent need for new treatment strategies to enhance patient outcomes (2).

Nucleosome- and spindle-associated protein 1 (NUSAP1) is a microtubule-associated protein essential for cell cycle regulation, particularly during mitosis, where it governs spindle assembly and stability to ensure proper chromosome segregation (6, 7). Recently, abnormal NUSAP1 expression has gained significant attention in various cancers. Studies have shown that elevated NUSAP1 expression is closely linked to heightened tumor cell proliferation, migration, and invasion, which is associated with a poor prognosis in various solid tumors, such as breast, prostate, and lung cancers (8, 9). In gliomas, NUSAP1’s role is becoming increasingly clear, with early studies suggesting that its elevated expression in glioma cells may drive malignant behaviors, such as invasiveness and resistance to apoptosis. Additionally, research into NUSAP1 as a potential therapeutic target is growing, indicating that modulating its expression and function could provide new treatment options for gliomas (10).

Single-cell sequencing technology, a state-of-the-art high-throughput analytical tool, has greatly advanced predictive, preventive, and personalized medicine (PPPM) (11). Single-cell sequencing facilitates an in-depth examination of individual cells across genomic, transcriptomic, and epigenomic dimensions, uncovering cellular heterogeneity, identifying rare disease-related cell populations, and elucidating their molecular profiles (12, 13). This approach provides essential insights for prediction and prevention in medical research. In PPPM, single-cell sequencing is widely used for early disease diagnosis and the development of personalized treatment plans. In oncology, for example, single-cell sequencing can uncover the complexity of the tumor microenvironment, identify resistant clones and potential therapeutic targets, and customize precise treatment regimens (14, 15). This technology also plays a crucial role in monitoring treatment responses and evaluating recurrence risks, aiding clinicians in optimizing therapeutic strategies and improving long-term survival rates. As a result, single-cell sequencing has become an essential tool in shifting from traditional treatment models to precision medicine, especially in cancer care.

In cancer immunology and oncology, the molecular mechanisms underlying tumor progression, immune evasion, and treatment resistance are of central importance for advancing therapeutic strategies (16, 17). Immunotherapy has emerged as a powerful approach to harness the body’s immune system to recognize and attack cancer cells, showing promise in various tumor types (18, 19). However, the effectiveness of immunotherapy, particularly in gliomas, remains limited due to the complex interplay of immune evasion mechanisms, tumor heterogeneity, and the molecular landscape of the tumor microenvironment (20, 21). Recent advances in single-cell technologies have revolutionized the study of these molecular and cellular mechanisms, enabling detailed profiling of immune cell populations, tumor cells, and their interactions within the tumor microenvironment (22, 23). Single-cell RNA sequencing, in particular, allows for the dissection of heterogeneity at the single-cell level, offering new insights into drug sensitivity, immune response, and treatment resistance in gliomas (24, 25). Nucleosome- and spindle-associated protein 1 (NUSAP1) has recently gained attention as a key player in cancer biology, with growing evidence supporting its role in cell cycle regulation, chromosomal instability, and tumor proliferation. In gliomas, elevated NUSAP1 expression has been associated with increased tumor aggressiveness, poor prognosis, and treatment resistance. Although NUSAP1 has not been extensively studied in the context of glioma immunology, its potential involvement in immune evasion and treatment sensitivity presents a promising area for exploration (26). As a targetable molecule, NUSAP1 could influence key immune regulatory pathways and modulate the tumor’s response to immunotherapy. Despite its emerging significance, the relationship between NUSAP1 and critical processes in cancer immunology and oncology, such as molecular mechanisms of immune regulation, immunotherapeutic responsiveness, and drug sensitivity, remains largely unexplored in gliomas. This gap in research highlights the need for further investigation into how NUSAP1 may contribute to immune suppression and treatment resistance in gliomas. Understanding the role of NUSAP1 in these processes could open new avenues for developing targeted therapies aimed at improving the efficacy of immunotherapy and overcoming drug resistance in glioma patients.

## 2 Methods

### 2.1 Data acquisition

Single-cell RNA sequencing (scRNA-seq) data was accessed from the Gene Expression Omnibus (GEO) database under accession number GSE141383 (https://www.ncbi.nlm.nih.gov/geo/), Bulk RNA-seq data was retrieved from The Cancer Genome Atlas (TCGA) through the official GDC portal (https://portal.gdc.cancer.gov/) (27).

### 2.2 Data filtering and processing

Each dataset was processed using the Seurat package (v4.3.0) in R (v4.2.2). Initially, potential doublets were excluded using the DoubletFinder package (v2.0.3), and low-quality cells were filtered out, maintaining cell quality within the following ranges: 300 < nFeature < 6000 and 500 < nCount < 100,000. Cells meeting these criteria were retained for further analysis. Additionally, cells were required to have less than 25% mitochondrial gene expression and less than 5% red blood cell gene expression to be included in subsequent analyses.

Next, the expression matrix was normalized, and the top 2000 highly variable genes (HVGs) were identified and standardized. PCA analysis was then performed on these genes. To address batch effects across samples, the Harmony package (v0.1.1) was utilized, selecting the top 30 principal components (PCs) for dimensionality reduction and clustering. Following this, UMAP was employed to project the results onto a two-dimensional plot, facilitating cell type identification. Relevant cell markers from the literature were used to annotate cell clusters, thereby identifying distinct cell types, and examining their distribution and proportions.

### 2.3 Enrichment analysis of differentially expressed genes

Differentially expressed genes (DEGs) for each cell type were identified using the “FindAllMarkers” function with default settings, employing the Wilcoxon rank-sum test. Genes expressed in over 25% of cells within clusters and exhibiting a log fold change (logFC) greater than 0.25 were selected. Enrichment analysis of differentially expressed genes (DEGs) was performed using the clusterProfiler package, with an emphasis on pathways related to each cell type as defined by Gene Ontology (GO) Biological Processes (BP).

### 2.4 Subpopulation analysis of glia/neuronal cells

To investigate the heterogeneity among glial and neuronal cells, we performed additional stratification. Following cell isolation, we identified the top 2,000 highly variable genes and proceeded with data normalization. The Harmony method was applied to minimize batch effects across samples. We selected the top 30 principal components (PCs) for downsampling and clustering, utilizing UMAP to project the data onto a two-dimensional map, facilitating the investigation of intercellular heterogeneity.

### 2.5 Malignant cell identification via inferCNV

Copy number variation (CNV) analysis was employed to distinguish malignant cells from non-tumor cells. By applying the inferCNV algorithm, we evaluated copy number variability across cell subpopulations, using vascular endothelial cells as a reference. Subpopulations with significant CNV alterations were classified as glioma cells.

### 2.6 Differential and enrichment analyses of cell subpopulations

Subsequently, the “FindAllMarkers” function was utilized to identify differentially expressed genes within each subpopulation using the Wilcoxon rank sum test. Following this, Gene Ontology Biological Process (GO-BP) enrichment analysis was performed with the clusterProfiler package.

### 2.7 Trajectory analysis

Three distinct software packages were employed to evaluate the differentiation progression across oligodendrocyte subpopulations. Initially, the cytoTRACE algorithm was utilized to assess the stemness levels within each subpopulation. Subsequently, differentiation trajectories were mapped using the Monocle R package (version 2.24.0), with the DDRTree algorithm employed to trace developmental progress along predetermined pathways. Finally, we conducted additional trajectory analysis during glioma differentiation using the Slingshot package. Minimum spanning trees (MSTs) were employed to infer cell lineages using the getLineages function, while the getCurves function was utilized to estimate temporal changes in gene expression within each lineage.

### 2.8 Cell communication analysis

To investigate the intricate intercellular communication among distinct cell subpopulations in GBM tumors and their interactions with the tumor microenvironment, we conducted a cross-talk analysis. The CellChat R package (version 1.6.1) and the CellChatDB.human reference database were utilized to examine ligand-receptor interactions. This analysis provided insights into cell-cell communication by evaluating signaling pathways and receptor-ligand interactions, revealing coordinated interactions among various cell types.

### 2.9 Prognostic modeling of glioma-associated cells

To assess the prognostic impact of glioma-associated cell subpopulations on patient survival, we identified key marker genes as predictive signatures for gliomas. We employed the survival R package to perform univariate Cox regression and Lasso regression for selecting significant prognostic genes. These genes were then incorporated into a prognostic model through multivariate Cox analysis. A risk score for each sample was computed using the following formula: risk score = (expression of gene 1 * coefficient 1) + (expression of gene 2 * coefficient 2) +… + (expression of gene n * coefficient n). Samples were categorized into high-risk and low-risk groups according to the median risk score. We then performed survival analysis to assess patient prognosis across these groups. The model’s accuracy was assessed using receiver operating characteristic (ROC) curves for 1, 3, and 5 years, employing the timeROC software package (version 0.4.0). Additionally, we examined the correlations between the prognostic genes, risk scores, and overall survival (OS).

### 2.10 Analysis of tumor-infiltrating immune cells

To thoroughly evaluate the immune microenvironment in patients, we employed CIBERSORT, ESTIMATE, and xCell to derive various immune-related scores. We assessed the levels of 22 immune cell types using the CIBERSORT algorithm, comparing high and low levels between groups. Additionally, we investigated the interactions among immune cells, risk scores, model genes, and overall survival (OS). We also analyzed TIDE scores and the expression of AODRA2A across different groups.

### 2.11 Differential and enrichment analyses of bulk genomic data

We performed differential analysis separately for high-risk and low-risk groups using the DESeq2 R package, applying a threshold of |logFC| > 2 and a p-value < 0.05. Additionally, we conducted Gene Ontology (GO), Kyoto Encyclopedia of Genes and Genomes (KEGG), and Gene Set Enrichment Analysis (GSEA) on the differentially expressed genes using the clusterProfiler package.

### 2.12 Somatic mutation analysis

Somatic mutation data from the TCGA repository were utilized to assess the mutational landscape of highly mutated genes relative to reference genes. Glioma samples were stratified into high and low groups based on the median tumor mutational burden (TMB). Kaplan-Meier survival analysis was subsequently conducted to compare survival outcomes between these groups. Additionally, we investigated the copy number variation (CNV) patterns of the targeted genes.

### 2.13 Drug sensitivity analysis

IC50 values for various chemotherapeutic agents were estimated using the pRRophetic R software (version 0.5). Sensitivity evaluations for these agents were then conducted across different categories.

### 2.14 Cell culture

U-251 and LN229 cell lines were cultured in Dulbecco’s Modified Eagle’s Medium (DMEM) supplemented with 10% fetal bovine serum (FBS) and 1% penicillin-streptomycin. Cells were maintained at 37°C in a humidified atmosphere with 5% CO2. For subculturing, cells were detached using trypsin-EDTA, counted, and reseeded at a density of 1 x 10^5 cells per flask. Cultures were routinely checked for mycoplasma contamination and passaged when they reached 80-90% confluency (28).

### 2.15 Cell transfection, lentivirus vector construction, and cell infection

Cells were seeded into 6-well plates at 70-80% confluence and transfected with plasmid DNA using Lipofectamine 3000 (RRID: AB_2572027, Invitrogen, Thermo Fisher Scientific) according to the manufacturer’s instructions. After 24 hours, the medium was replaced with fresh medium, and the sequences used for transfection are listed in **Supplementary Table 1**. Lentiviral vectors were constructed by cloning target genes into a lentiviral expression plasmid (e.g., pLenti6.3/V5-DEST, RRID: Addgene_17452). The plasmid was co-transfected with packaging plasmids (pMD2.G, RRID: Addgene_12259 and psPAX2, RRID: Addgene_12260) into HEK293T cells (RRID: CVCL_0063, ATCC) using Lipofectamine 3000. Forty-eight to seventy-two hours post-transfection, viral supernatants were collected, filtered through a 0.45 µm membrane, and concentrated using a lentivirus concentration kit (Lenti-X Concentrator, Clontech, Takara Bio, catalog number 631231). Target cells were infected with lentivirus in the presence of 8 µg/mL polybrene (Sigma-Aldrich, catalog number 107689). After 24 hours, the virus-containing medium was replaced with fresh growth medium. Infection efficiency was assessed 48 hours later using fluorescence microscopy or flow cytometry (29).

### 2.16 RT-qPCR analysis

Total RNA was extracted from cells using the RNeasy Mini Kit (Qiagen, RRID: AB_2650242) according to the manufacturer’s protocol. RNA was subsequently reverse transcribed to complementary DNA (cDNA) using the PrimeScript RT Reagent Kit (Takara, RRID: AB_10050579). Quantitative PCR (qPCR) was performed using SYBR Green Master Mix (Applied Biosystems, RRID: AB_2733300) on a real-time PCR system (Applied Biosystems 7500, RRID: AB_2647999). The cycling conditions consisted of an initial denaturation at 95°C for 10 minutes, followed by 40 cycles of 95°C for 15 seconds and 60°C for 1 minute. Gene expression levels were normalized to GAPDH (GenBank accession no. NM_002046), and relative expression was calculated using the ΔΔCt method. Statistical analysis was conducted using appropriate software (e.g., GraphPad Prism), and p-values < 0.05 were considered statistically significant. Primer sequences are listed in **Supplementary Table 1**.

### 2.17 Cell counting kit-8 assay

Cell viability was assessed using the Cell Counting Kit-8 (CCK-8, Dojindo). Cells were seeded in 96-well plates at a density of 2 x 10^3^ cells per well and allowed to adhere overnight. After treatment, 10 μL of CCK-8 solution was added to each well, and cells were incubated for 2 hours at 37°C. The absorbance at 450 nm was measured using a microplate reader. Data were normalized to control wells, and statistical analysis was performed with p-values < 0.05 considered significant.

### 2.18 Colony formation assay

A cohort of 1000 cells was transfected and incubated in 6-well plates for approximately 14 days under laboratory conditions. After this two-week period, cellular clones were visually inspected without assistance. Subsequently, the cells were washed and fixed with a 4% paraformaldehyde (PFA) solution for 15 minutes. Following fixation, the cells were stained with crystal violet (Solarbio, China) for 20 minutes and allowed to air-dry at room temperature. Finally, cell quantification per well was conducted in the study.

### 2.19 Wound healing assay

Wound healing assays were conducted by creating a uniform scratch in a confluent cell monolayer using a sterile pipette tip. Cells were then washed to remove debris and incubated in fresh medium. Wound closure was monitored at 0 and 24 hours using a phase-contrast microscope. Images were captured, and the wound area was measured using ImageJ software. The percentage of wound closure was calculated relative to the initial wound area, and statistical analysis was performed with p-values < 0.05 considered significant.

### 2.20 Transwell migration assay

Cell migration was assessed using a Transwell migration assay. Cells were suspended in serum-free medium and seeded into the upper chamber of Transwell inserts with an 8 μm pore size (Corning). The lower chamber was filled with medium containing 10% fetal bovine serum as a chemoattractant. After 24 hours of incubation at 37°C, non-migrated cells on the upper membrane were removed with a cotton swab, and migrated cells on the lower membrane were fixed with 4% paraformaldehyde and stained with crystal violet. The number of migrated cells was counted under a light microscope, and statistical analysis was performed with p-values < 0.05 considered significant.

### 2.21 Statistical analysis

Biological analyses were performed using R software version 4.1.3, while experimental data analysis was conducted with GraphPad Prism version 8.0. Mean values and standard deviations were derived from three independent experiments. Comparisons between two groups were made using Student’s t-test, whereas one-way ANOVA followed by Tukey’s *post hoc* test was employed for comparisons among multiple groups. Statistical significance was indicated as follows: *p<0.05, **p<0.01, ***p<0.001.

## 3 Results

### 3.1 Key cell types involved in glioma progression identified via snRNA sequencing

**Figure 1:**
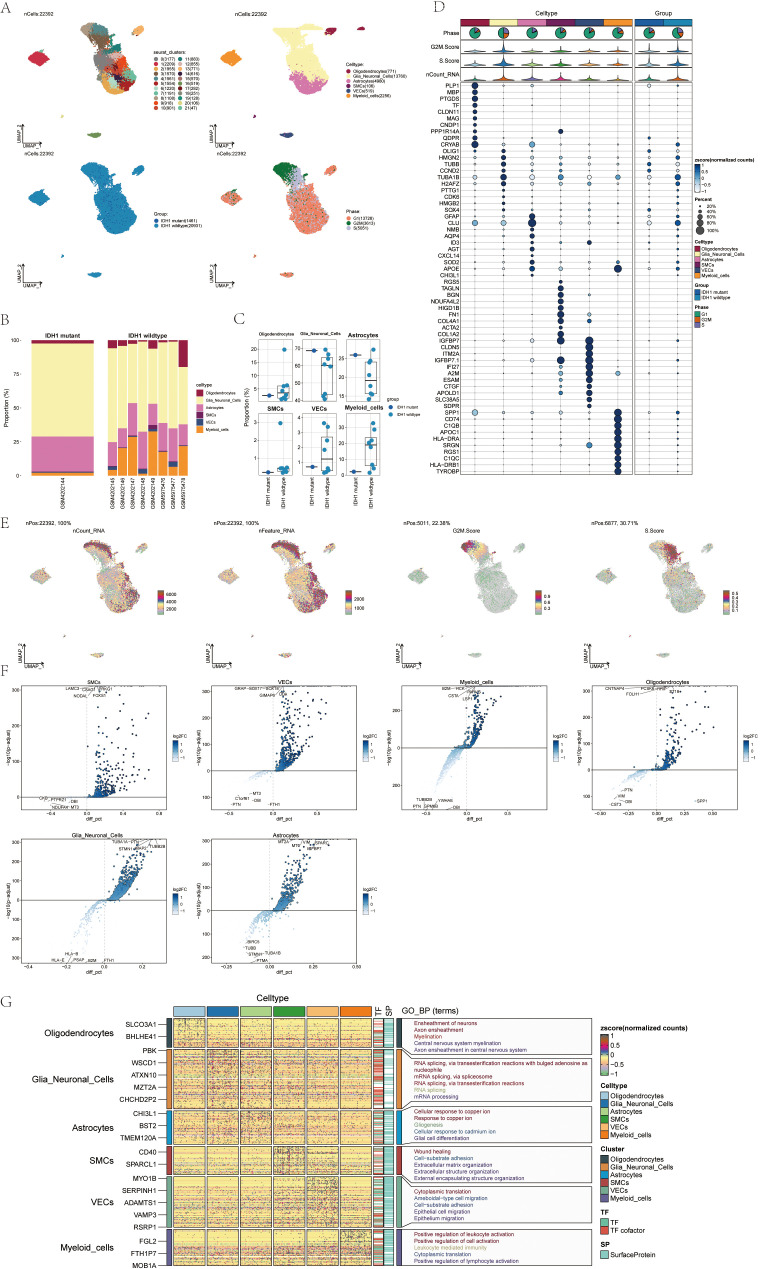
snRNA sequencing reveals major cell types during GBM progression **(A)** UMAP projections of 22392 aggregate single cells from 9 patients showing the composition of different cell types in human gliomas. UMAP projections are shown by cluster numbers, by cluster assignment, by groups, and by cell cycle phases. **(B)** Bar graph showing the percentage of the 6 cell types in the IDH1 group versus the IDH1 wildtype group. **(C)** Box line plot depicting the percentage of the 6 cell types in the GBM group versus the IDHR132H WT GBM group. **(D)** Bubble plot showing differential expression of the Top10maker genes in glioma cells in different cell types. **(E)** The UMAP plot showcases the distribution of the following relevant features: nCount_RNA, nFeature_RNA, G2M.score, and S.score. **(F)** Volcano plot demonstrating differential gene expression in 6 cell types. **(G)** GO-BP enrichment analysis revealing biological processes associated with the 6 cell types.

Single-nucleus RNA sequencing (snRNA-seq) was conducted on tumor samples from nine glioma patients to investigate the cellular diversity within these specimens. After quality control and filtering, we analyzed 22,392 cells through dimensionality reduction clustering, identifying 22 clusters corresponding to six different cell types: oligodendrocytes (771), glial neuronal cells (13,760), astrocytes (4,980), smooth muscle cells (106), vascular cells (519), and myeloid cells (2,256) (**Figure 1A**, upper). Among the 22,392 cells analyzed, 14,611 exhibited the IDH1 mutation, while 20,931 were IDH1 wild-type (**Figure 1A**). UMAP plots illustrated the distribution of these six cell types across various cell cycle phases: 5,051 cells in the S-phase, 13,728 in the G1 phase, and 3,613 in the G2M phase (**Figure 1A**, bottom).

A bar chart was employed to depict the distribution of the six cell types within a cohort consisting of eight patients with IDH1 wild-type lesions and one patient with IDH1 mutant lesions (**Figure 1B**). This chart highlights the heterogeneity of cell types among the glioma patients. Box plots further emphasized the distinct variations among these six cellular phenotypes across the different cohorts (**Figure 1C**). Marker gene expression varied among the six cell types identified in the tumor samples, with the top 10 markers for each cell type displayed in bubble plots (**Figure 1D**).

RNA characteristics, including nCount_RNA, nFeature_RNA, G2M score, and S score, were visualized through UMAP plots (**Figure 1E**). Word clouds displayed Gene Ontology Biological Process (GO-BP) enriched pathway terms across the cell types while volcano plots highlighted distinct genes among the six cell types (**Figure 1F**). Heatmaps presented GO-BP enrichment analysis results for uniquely expressed genes within the six cellular phenotypes (**Figure 1G**).

### 3.2 Intracellular heterogeneity in glia-neuronal cells

**Figure 2:**
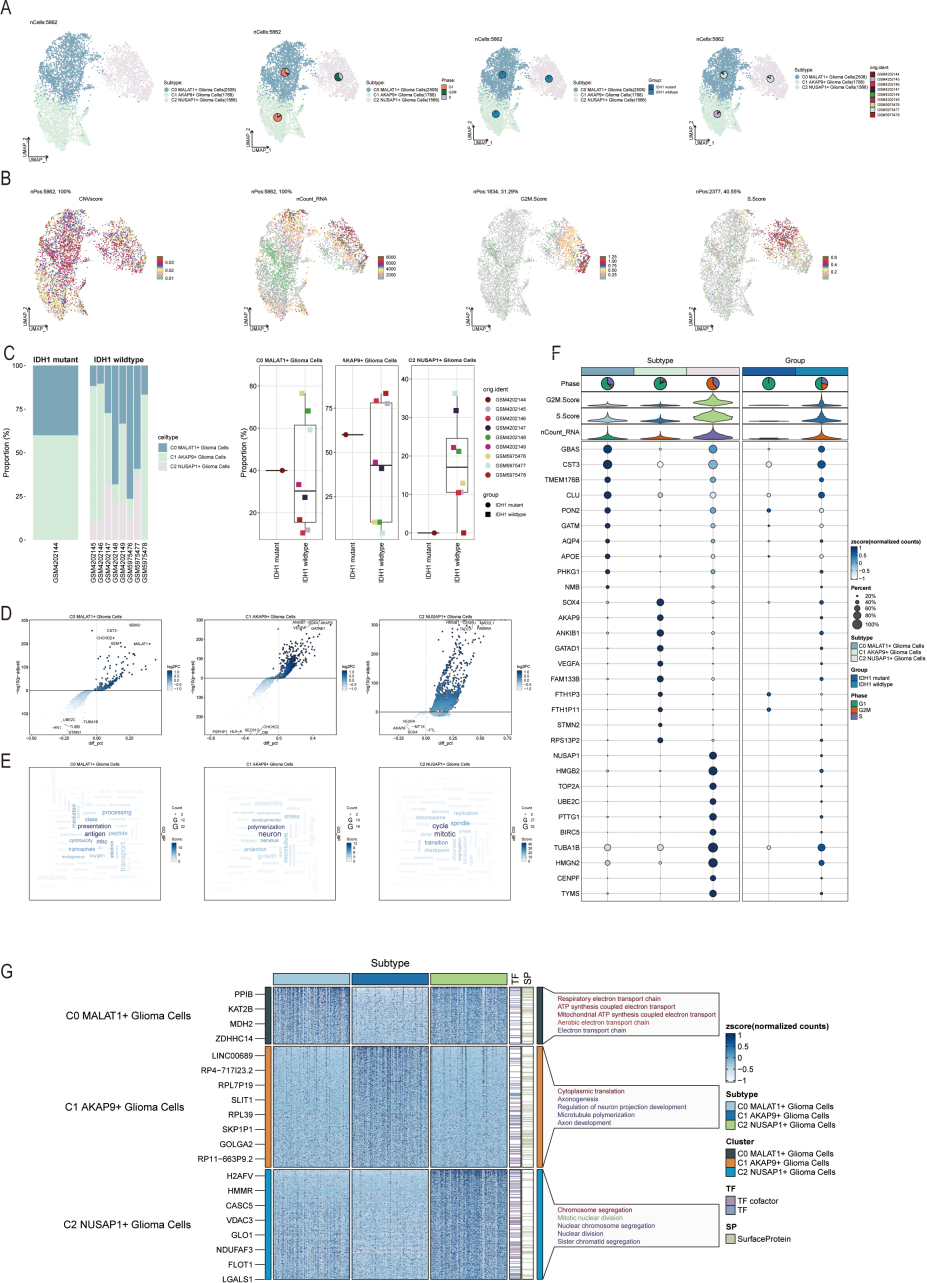
Visualization of Glia-Neuronal-cells cell subpopulations **(A)** UMAP diagram demonstrating the 3 cell subpopulations of tumor cells in glioma patients and the number of cells in each subpopulation (top left); UMAP diagram demonstrating the percentage of different cell cycles in the 3 cell subpopulations (top right); UMAP diagram demonstrating the distribution of the IDH1 mutant group versus the IDH1 wildtype group in the 3 cell subpopulations (bottom left); and UMAP diagram demonstrating the patient origin of the 3 cell subpopulations (lower right). **(B)** UMAP plot visualizing the pertinent features of the 3 cell subpopulations: CNVscore, nCount_RNA, G2M.score, S.score. **(C)** Bar graph demonstrating the percentage of the 3 cell subpopulations in the IDH1 mutant group versus the IDH1 wildtype group (left); box line graph depicting the percentage of the 3 cell subpopulations in the IDH1 mutant group versus the IDH1 wildtype group (right). **(D)** Volcano plot demonstrating the expression of differential genes in the 3 cellular subpopulations. **(E)** Word cloud graph demonstrating gene pathway enrichment in the 3 cell subpopulations. **(F)** Bubble plot showing differential expression of Top10maker genes in 3 cell subpopulations. **(G)** GO-BP enrichment analysis demonstrating biological processes associated with the 3 cell subpopulations.

To analyze glia-neuronal cells for malignancy, we applied an inferred CNV algorithm to identify malignant cells at the single-cell level. Based on inferred copy number variations (CNV), cells with elevated CNV levels were classified as glioma cells (**Supplementary Figure 1**) revealing three subpopulations: C0 MALAT1+ glioma cells (2,508), C1 AKAP9+ glioma cells (1,788), and C2 NUSAP1+ glioma cells (1,566) (**Figure 2A**). We examined the distribution of cell cycle stages, subsets, and lineages across these three subgroups.

UMAP representations were used to visualize the CNV score, nCount_RNA, S score, G2M score, and other relevant attributes of the three cellular subgroups (**Figure 2B**). Additionally, the distribution of these three subtypes was visually examined across eight cases with IDH1 wild-type lesions and one case with an IDH1 mutant lesion (**Figure 2C**, left). The C2 NUSAP+ glioma subpopulation was predominantly found in patients with SF12264. Box plots showed varying proportions of the three subpopulations across the groups (**Figure 2C**, right). Volcano plots highlighted differentially expressed genes in each of the three subpopulations (**Figure 2D**). Word clouds displayed enriched Gene Ontology Biological Process (GO-BP) pathway terms within these subgroups (**Figure 2E**). Marker genes with differential expression in the three subpopulations were visualized using bubble plots and heatmaps (**Figure 2F**), A heatmap was generated to illustrate the results of GO-BP enrichment analysis for the distinct gene sets in the three subgroups (**Figure 2G**).

### 3.3 Pseudotemporal analysis of glial and neuronal cell subpopulations using CytoTRACE and monocle

**Figure 3:**
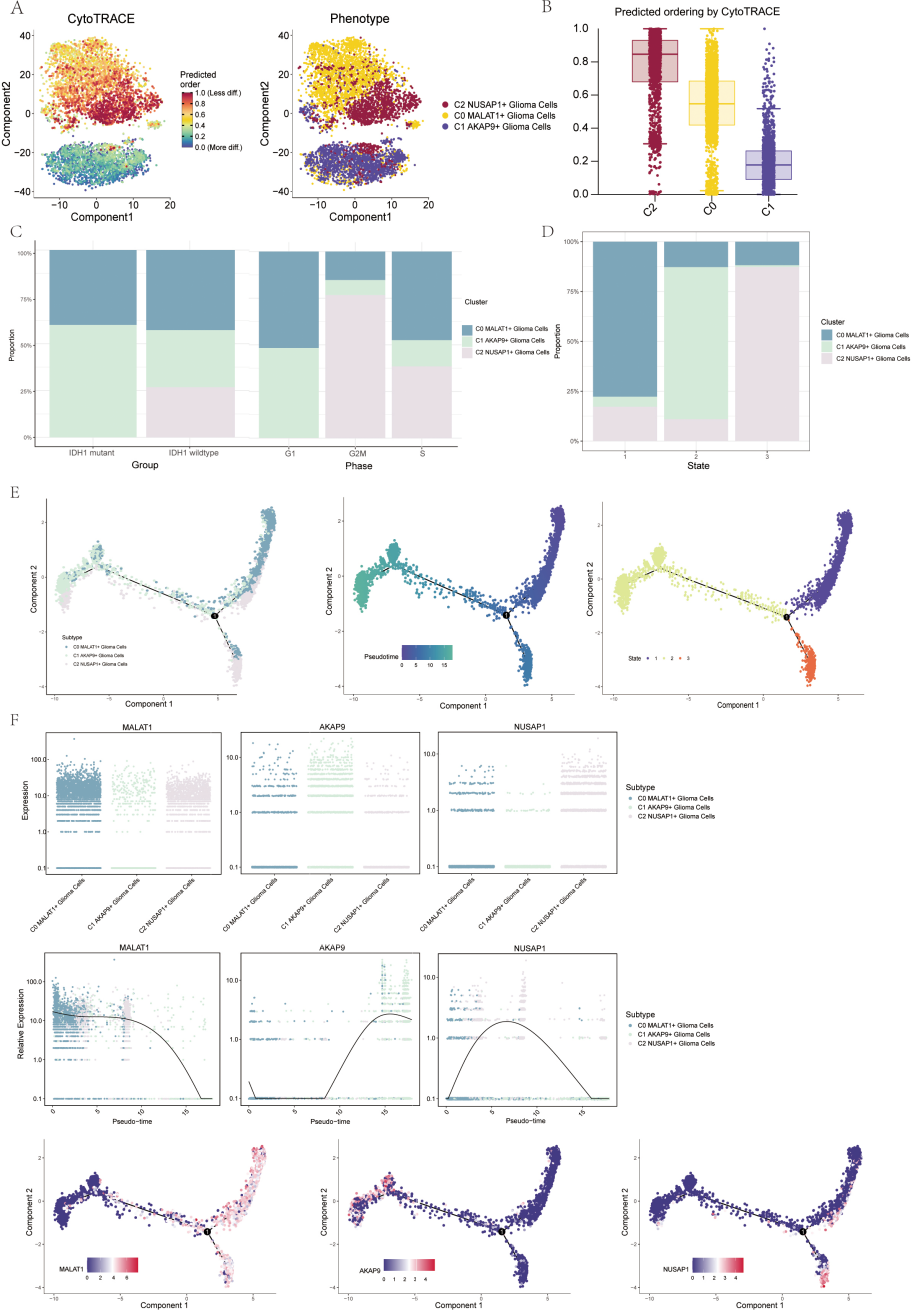
Visualization of proposed time series analysis of 3 glioma cell subpopulations by cytotrace and monocle **(A)** The differentiation of glioma cell subpopulations is analyzed using cytotrace. The color can represent the level of differentiation. The figure on the right represents the cytotrace results displayed according to different glioma cell subpopulations. The colors represent different cell subpopulations. **(B)** Box line plot showing the visualization results of cytotrace analysis of glioma cell subpopulations. **(C)** The occupancy of relevant features in different pseudotime stages of 3 cell subpopulations was visualized": the occupancy of 3 cell subpopulations in the IDH1 mutant group versus the IDH1 wildtype group (left); the occupancy of 3 cell subpopulations in different cell cycles (right). **(D)** Bar charts illustrating the proportions of different pseudotime stages (state1-state3) within the three cell subgroups. **(E)** Demonstrating the derivation process of glioma cell subpopulation. Left: UAMP plot of the proposed temporal trajectory showing the 3 cell subpopulations; Middle: UMAP plot showing the pseudotime trajectory of glioma cell subpopulation, starting from the upper right and dividing into two branches, one of which differentiates downward and the other to the left; Right: UMAP plot showing the distribution of 3 states on the proposed temporal trajectory plot. **(F)** Scatter plot showing the changes of 3 cell subpopulations of glioma cell subpopulation; proposed chronological sequence UMAP plot showing the changes of the cell subpopulations corresponding to the 3 named genes with the proposed chronological sequence; and the expression of the 3 named genes of cell subpopulations (MALAT1, AKAP9, NUSAP1) on the pseudotime trajectory.

To explore the differentiation and developmental trajectories of glioma cell subpopulations, we conducted CytoTRACE analysis (**Figure 3A**), revealing a differentiation pattern from C1 to C0 and then to C2 (**Figure 3B**). A bar graph illustrates the distribution of the three cell subpopulations across IDH1 mutant and IDH1 wildtype cohorts (**Figure 3C**, left). Notably, the C2 NUSAP1+ glioma cell subpopulation was exclusive to the IDH1 wildtype group. This finding provides a starting point to explore the relationship between the IDH1 mutant and wildtype groups. Additional bar graphs illustrated the distribution of the three cell subpopulations across different stages of the cell cycle (**Figure 3C**, right). nd to show the changes in cell percentages during different stages of trajectory differentiation (**Figure 3D**). In state 1, the C0 MALAT1-expressing glioma cells were the most prevalent. In state 2, C1 AKAP9-expressing glioma cells dominated, while in state 3, C2 NUSAP1-expressing glioma cells were the most abundant.

To gain further insights into glioma development, we conducted additional trajectory analysis of the three cellular subtypes using monoclonal techniques (**Figure 3E**). The three cellular subtypes displayed a continuous distribution along the pseudotemporal trajectory, eventually diverging at a branching point. The trajectory commenced in the upper right quadrant and advanced to the differentiation point designated as state 1. At this point, the lineage split: one branch continued caudally, corresponding with state 3, while the other moved leftward, aligning with state 2. Chronologically, the C1 AKAP9+ glioma cell subpopulation appears to represent the early stage of tumorigenesis. As tumorigenesis progresses, this subpopulation differentiates into other subtypes, eventually becoming C0 DOCK5+ or C2 NUSAP+ glioma cells. We identified the genes associated with the three cellular subpopulations and mapped their dynamic trajectories using pseudo-time series scatter plots, UMAP plots, and pseudo-scatter plots. The analysis revealed that the C1 cell subset, marked by the AKAP9 gene, was predominantly present early in the temporal sequence, while the C2 subset, characterized by the NUSAP1 gene, was more prevalent towards the end of the pseudotemporal series (**Figure 3F**).

3.4 Slingshot Analysis of Pseudotemporal Trajectories of Glioma Cell Subpopulations

**Figure 4:**
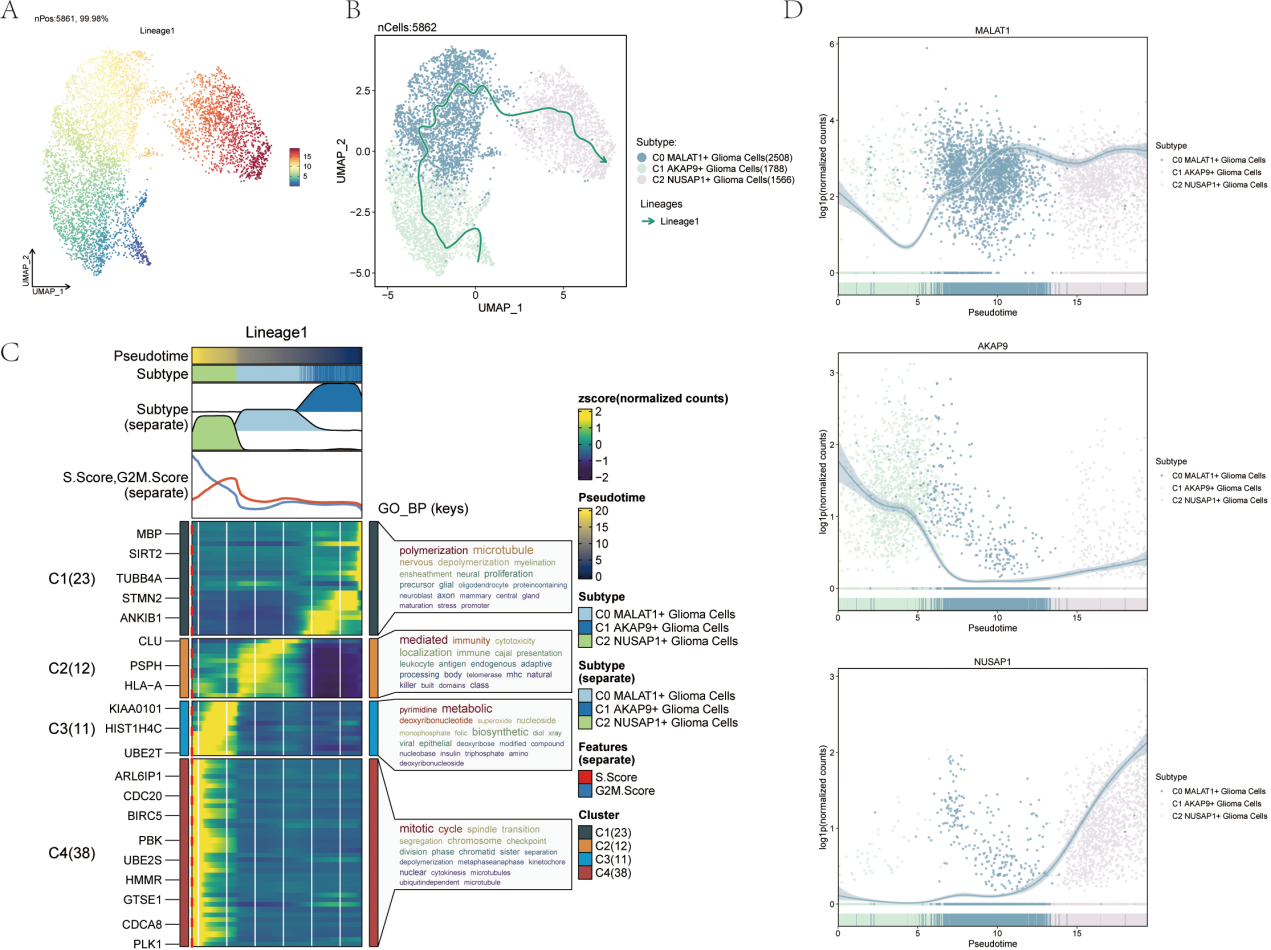
Slingshot analysis of the pseudotime trajectories of glioma cell subpopulations **(A)** UMAP plot demonstrating the change of Lineage1 with the fitted temporal order; **(B)** UMAP plot demonstrating the differentiation trajectory of Lineage1 on the fitted temporal order (right). **(C)** GO- BP enrichment analysis demonstrating the biological processes corresponding to the proposed chronological trajectory of glioma cell subpopulations. **(D)** Scatterplot demonstrating the trajectories of the named genes of the three cell subpopulations of glioma cell subpopulations obtained after slingshot visualization.

We further analyzed the differentiation trajectories of the three glioma cell subpopulations (C0 to C2) using the slingshot method. These subpopulations were consistently distributed along the temporal axis, forming a lineage (**Figure 4A**). Spectrum 1 illustrates the progression from C1 to C0, followed by a shift to C2 (**Figure 4B**). To understand the biological mechanisms underlying these pseudotemporal trajectories, We conducted enrichment analysis for GO-BP. The C1 subpopulation was associated with processes like polymerization and microtubule dynamics. C2 was linked to signaling mediation and immune responses, while C3 was connected to pyrimidine metabolism, and C4 to mitotic processes (**Figure 4C**). Scatter plots illustrated the distribution of different cell subpopulations along Spectrum 1, highlighting their unique differentiation patterns over pseudotime (**Figure 4D**).

### 3.4 CellChat analysis of cell-cell interactions and PTN signaling pathway visualization

**Figure 5:**
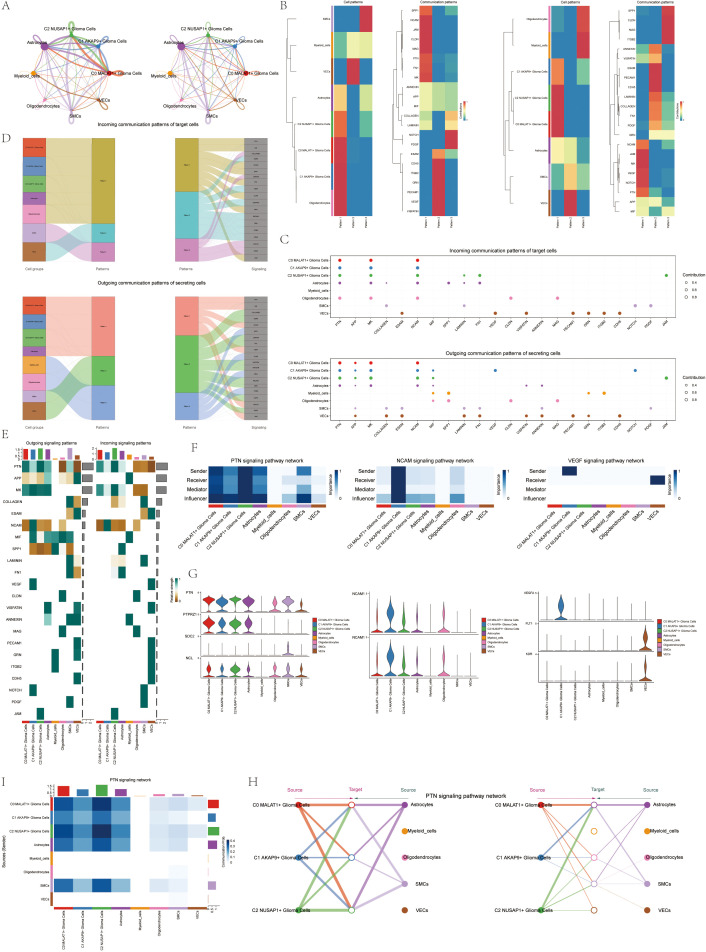
CellChat results presentation **(A)** Circle plot showing the number (left) and strength (right) of interactions between all cells. **(B)** Heatmap showing pattern recognition of outcoming cells in all cells (left), and pattern recognition of incoming cells (right). **(C)** outcoming contribution bubble plots and incoming contribution bubble plots showing the expression of cellular communication patterns between each cell subpopulation and other cells in the glioma cell subpopulation. **(D)** Mulberry diagram showing cellular communication patterns between all cells. Top: incoming Mulberry diagram, bottom: outcoming Mulberry diagram. **(E)** Heatmap showing afferent and efferent signal intensities of all cell interactions. **(F)** Heatmap showing PTN, NCAM, and VEGF signaling pathway network centrality scores. **(G)** Violin diagram of cellular interactions in the PTN, NCAM, and VEGF signaling pathways. **(H)** Hierarchical diagram of glioma cell subpopulations interacting with other cells in the PTN signaling pathway. **(I)** Interaction of cells in the PTN signaling pathway shown by heatmap.

To better understand complex cellular responses, we aimed to explore intercellular dynamics and ligand-receptor communication networks. Using CellChat analysis, we constructed intercellular communication networks involving various cell types, such as glioma subpopulations, oligodendrocytes, myeloid cells, astrocytes, smooth muscle cells, and vascular cells. We quantified interaction frequencies by measuring connection thickness and assessed interaction intensities based on line weights (**Figure 5A**). To investigate the coordination of various cell populations and signaling pathways, we used CellChat’s non-negative matrix decomposition technique for pattern recognition. Our analysis revealed communication patterns connecting cell populations as either signal transmitters (outbound) or recipients (inbound). Using CellChat’s gene expression tool, we further explored these cellular interactions and signaling pathways. We first linked hypothesized communication patterns to secretory cell cohorts to clarify efferent signaling modalities. Then, we correlated these patterns with secretory cell populations. We identified three communication modes, each associated with a specific cell type: mode 1 (glioma subpopulations), mode 2 (vascular endothelial cells), and mode 3 (oligodendrocytes and myeloid cells) (**Figure 5B**). For glioma efferent signaling, mode 1 was characterized by pathways such as PTN and NCAM. In contrast, glioma afferent signaling mainly involved mode 1 communication patterns, including pathways like VEGF, PTN, and JAM (**Figure 5D**). Employing CellChat’s pattern recognition methodology, we quantitatively analyzed the ligand-receptor interactions within gliomas to pinpoint key signaling pathways associated with the three cell subtypes. In gliomas, each cell variant functions as both a sender and receiver in the communication network, releasing various cytokines or ligands and responding to signals from other cells (**Figure 5C**).

We created a heatmap to visualize the intensity of afferent and efferent signals across all cell interactions, specifically focusing on participation in the PTN signaling pathway (**Figure 5E**). Utilizing a centrality measurement algorithm, we classified cell types according to their roles as mediators and influencers in intercellular communication. The Glioma C2 subset, characterized by NUSAP expression, was identified as a pivotal player in the PTN signaling cascade. Additionally, we found that the NCAM signaling pathway, involved in cell adhesion, and the VEGF pathway, associated with angiogenesis, were particularly prominent in the C1 AKAP9+ Glioma subpopulation (**Figure 5F**). Violin plots highlight cellular interactions, showing the NUSAP+ Glioma subpopulation in the C2 group with elevated activity in the PTN signaling cascade. In contrast, the AKAP9+ Glioma subset in the C1 group was notably involved in the NCAM and VEGF signaling pathways (**Figure 5G**). We identified all eight cell types within glioma tissues as origins of the PTN signaling cascade. The three glioma subtypes, along with other cell types, were considered potential targets, highlighting their correlations within the PTN signaling pathway in a hierarchical plot. The findings suggest that, except for myeloid cells, oligodendrocytes, and vascular endothelial cells (VECs), various cell types act as signaling mediators within the PTN cascade. A heatmap displaying the complex network of cell-cell interactions is shown in **Figures 5I, H**.

### 3.5 Development and validation of a prognostic model

**Figure 6:**
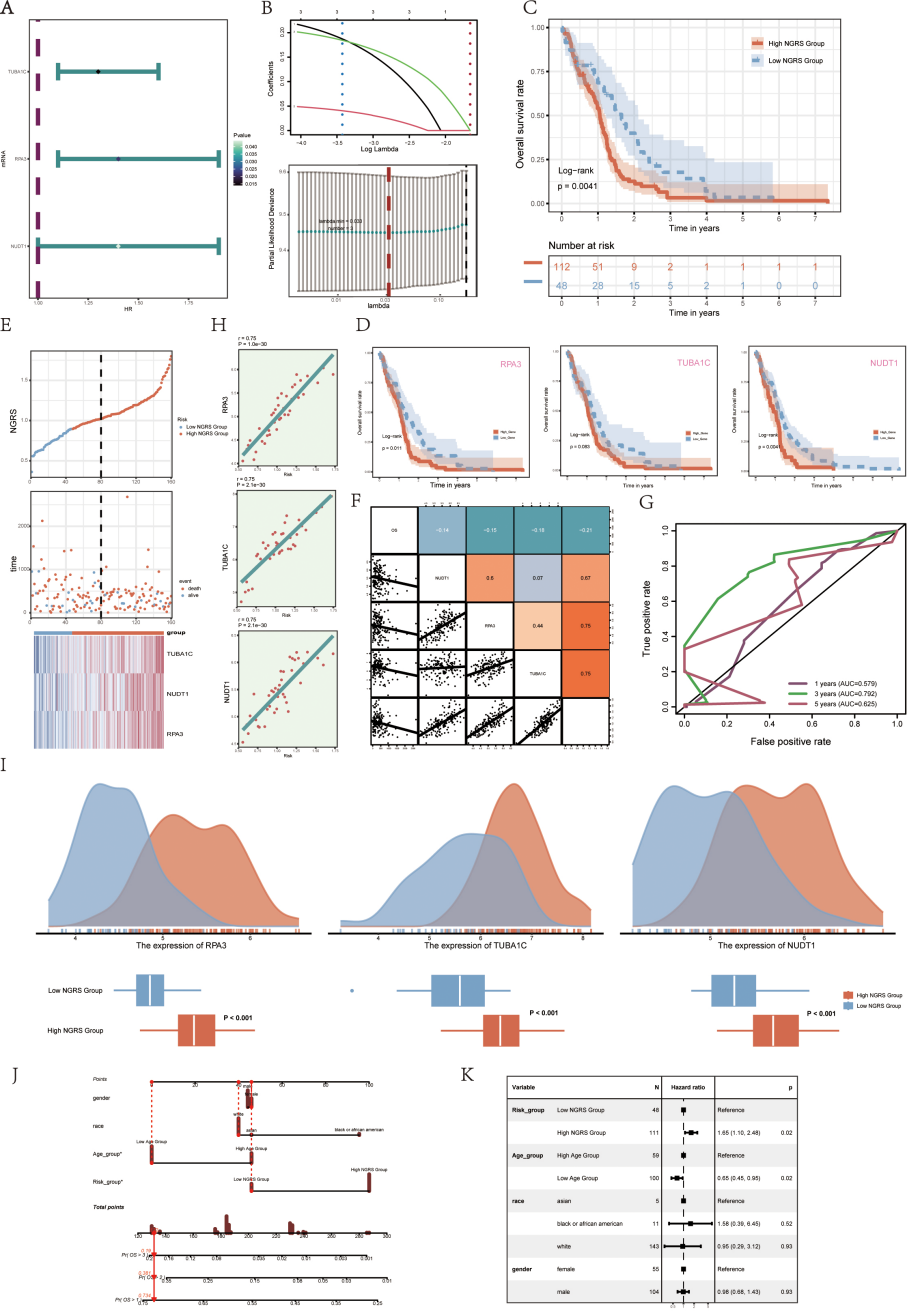
Modeling of prognosis associated with the C2 NUSAP+ glioma score **(A)** Forest plot showing univariate Cox analyses of the genes comprising the C2 NUSAP+ glioma score. Null line HR=1, HR<1 protective factor, HR>1 risk factor. **(B)** The three genes comprising the C2 NUSAP+ glioma score were screened by lasso regression (upper panel); Lambda plot of the genes comprising the C2 NUSAP+ glioma score (lower panel). **(C)** Survival analysis of the 3 genes screened for constituting the C2 NUSAP+ glioma score in the C2 NUSAP+ glioma high and low subgroups. **(D)** Survival analysis plot for the 3 genes comprising the C2 NUSAP+ glioma high and low subgroups. **(E)** Curve plots showing risk scores for C2 NUSAP+ glioma high and low subgroups (upper panel); scatter plots showing changes in survival status between C2 NUSAP+ glioma high and low subgroups (middle panel); and heat maps showing gene expression in C2 NUSAP+ glioma high and low subgroups with color scales based on normalized data (lower panel). Blue color indicates the C2 NUSAP+ glioma low subgroup and red color indicates the C2 NUSAP+ glioma high subgroup. **(F)** Correlation analysis between C2 NUSAP+ glioma score, overall survival (OS), and genes used for modeling. Orange indicates a positive correlation, blue indicates a negative correlation, and color shades indicate high or low correlation. **(G)** AUC scores at 1, 3, and 5 years are shown in ROC plots.AUC (1 year): 0.579, AUC (3 years): 0.792, AUC (5 years): 0.625. **(H)** Scatterplot illustrating the correlation analysis of the genes comprising the C2 NUSAP+ glioma score with the C2 NUSAP+ glioma score. **(I)** Peak and box plots illustrate the variations in the expression of the three genes comprising the C2 NUSAP+ glioma score between the high and low score groups of C2 NUSAP+ glioma. **(J)** Combined plot predicting 1-, 3-, and 5-year overall survival (OS) based on age, C2 NUSAP+ glioma high and low score subgroups, and stage. **(K)** Forest plot displaying multivariate Cox regression analysis of C2 NUSAP+ glioma score versus other clinical factors. In a null line, HR=1. An HR<1 is considered a protective factor, while an HR>1 is seen as a risk factor. *p<0.05.

To assess the clinical significance of the identified cell types, we performed a univariate Cox analysis on the top 100 marker genes within the C2 NUSAP+ Glioma subgroup. This analysis revealed three genes—RPA3, TUBA1C, and NUDT1—that are associated with patient outcomes (**Figure 6A**). To address multicollinearity within the gene pool, we used lasso regression to refine the selection, identifying three key genes crucial to the NUSAP+ Glioma scoring system. Lambda plots validated the robustness of this gene subset (**Figure 6B**). Patients were categorized into two groups based on their expression levels of the selected genes: high and low NUSAP+ Glioma score groups. Survival analysis indicated that individuals in the low NUSAP+ Glioma score group experienced superior survival outcomes compared to those in the high NUSAP+ Glioma score group (**Figure 6C**). Survival analysis was conducted for the three genes that make up the NUSAP+ Glioma score model (**Figure 6D**). The results consistently demonstrated that elevated expression levels correlated with poorer survival, whereas reduced expression levels were associated with improved prognostic outcomes, thereby affirming their role as risk factors. The NUSAP+ Glioma score for each patient in the TCGA-GBM cohort was determined by integrating gene expression levels with their corresponding regression coefficients. Subsequently, patients were categorized into high and low score groups based on the median value. A higher NUSAP+ Glioma score was associated with reduced survival times. Expression levels of the three genes in the model are depicted in **Figure 6E**. Correlation analysis revealed an inverse relationship between overall survival (OS) and both the NUSAP+ Glioma score and the three genes. These relationships are visually represented in scatter plots (**Figure 6F**). ROC curves were utilized to evaluate the predictive accuracy of the NUSAP+ Glioma score for 1-year, 3-year, and 5-year survival, yielding AUC values of 0.579, 0.792, and 0.625, respectively (**Figure 6G**). Scatter plots displayed the genetic factors correlated with NUSAP+ Glioma scores (**Figure 6H**), and **Figure 6I** highlighted differences in gene expression levels between high and low NUSAP+ Glioma score groups. Multifactorial Cox regression analysis was performed to determine if the NUSAP+ Glioma score serves as an independent prognostic factor. This analysis incorporated variables such as age, race, T stage, N stage, M stage, and the NUSAP+ Glioma score, revealing the latter as a significant independent predictor of prognosis in glioma patients (p < 0.05) (**Figure 6K**). Additionally, a column chart was generated to integrate clinical and pathological risk factors with cell type characteristics, utilizing age, race, and T stage. This chart provides an effective prediction of patient survival probabilities at 1, 3, and 5 years (**Figure 6J**).

### 3.6 Immune infiltration differences between high and low NUSAP+ Glioma score groups

**Figure 7:**
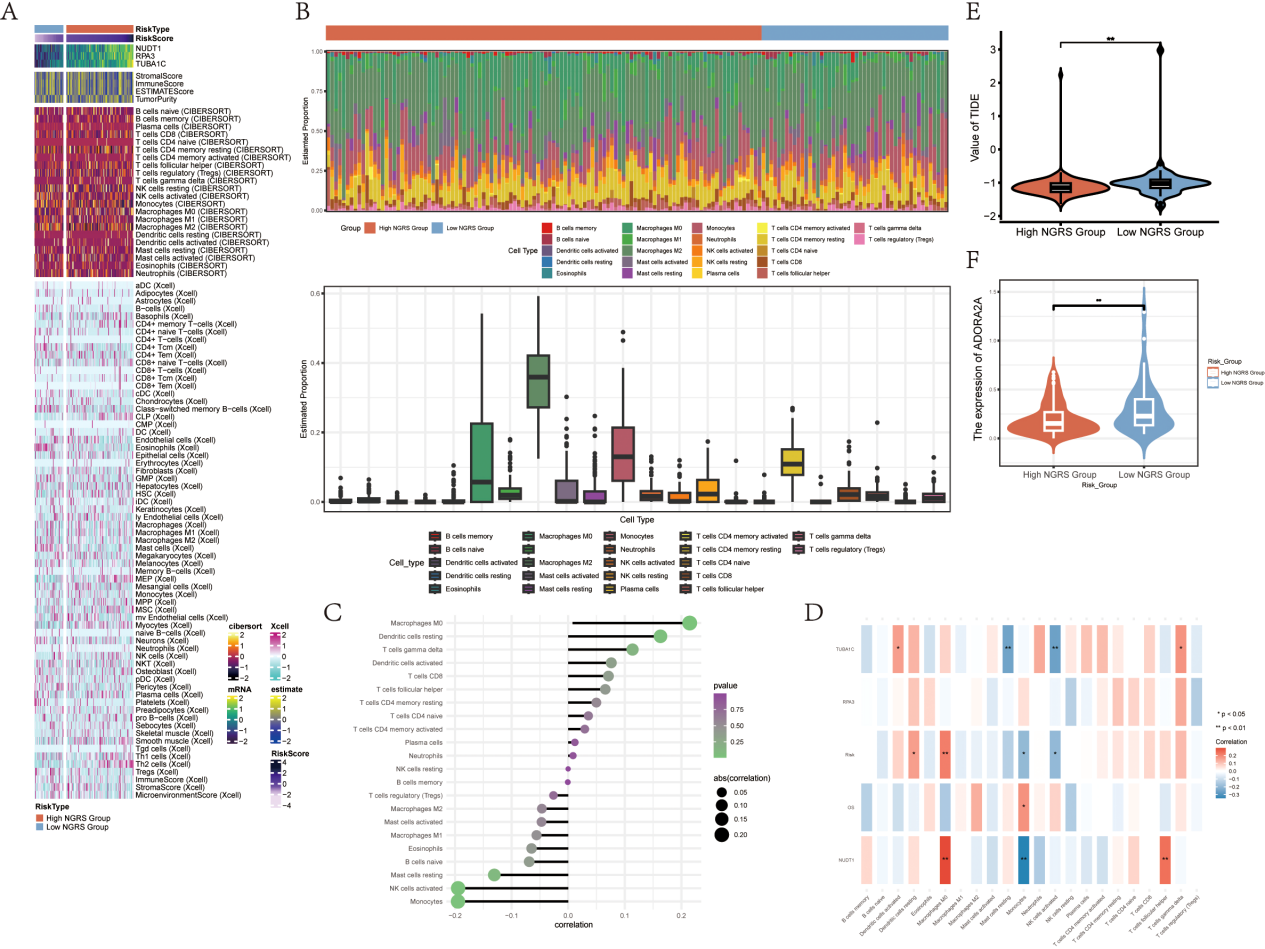
Differential Analysis of Immune Infiltration in High and Low Subgroups of C2 NUSAP+ Gliomas **(A)** Heatmap showing the expression of various immune scores in high and low subgroups of C2 NUSAP+ gliomas. **(B)** Immune infiltration superimposed bar graph (upper panel); box-and-line plot showing the expression of 22 immune cells in gliomas (lower panel). **(C)** Lollipop plot showing the correlation between immune cells and C2 NUSAP+ glioma scores. **(D)** Correlation between immune cells and C2 NUSAP+ glioma score genes is shown as a bar graph with a heat map. *P ≤ 0.05, **P ≤ 0.01; ***P≤ 0.001 indicates a significant difference, and NS indicates a non-significant difference. **(E)** Violin plot showing the high and low TIDE values in the high and low subgroups of C2 NUSAP+ gliomas. **(F)** Violin plots showing the expression of AODRA2A between different groups of C2 NUSAP+ gliomas. *p ≤ 0.05, **p ≤ 0.01; ***p ≤ 0.001 indicates a significant difference, ns indicates a non-significant difference.

To investigate immune infiltration in gliomas and assess differences in immune cell populations between high and low NUSAP+ Glioma score groups, we utilized heatmaps to visualize the variations in immune cell infiltration within each group (**Figure 7A**). We next evaluated immune cell infiltration in glioma patients by analyzing data from the TCGA repository using the CIBERSORT computational tool. Heatmaps were employed to display the distribution of 22 distinct immune cell types identified in the samples (**Figure 7B**). Bar graphs were used to depict the relationships between immune cell types and glioma subpopulation markers. The NUSAP+ Glioma scores showed a positive correlation with M0 macrophages, resting dendritic cells, and other immune cells, whereas they were negatively correlated with activated NK cells, monocytes, and additional cell types (**Figure 7C**). Various methods of immune cell content assessment were used to compare and summarize the associations between the three genes under study and immune cells. These relationships were illustrated using heatmaps, where positive correlations were represented in red and negative correlations were depicted in blue (**Figure 7D**). Violin plots demonstrated immune dysfunction and tumor rejection across both high and low scoring groups, with a significantly elevated TIDE score observed in the low scoring group compared to the high scoring group (**Figures 7E, F**). The expression of ADORA2A was present in tumors from both groups, but it was notably lower in the high NUSAP+ Glioma score group relative to the low scoring group.

### 3.7 Differential and enrichment analysis

**Figure 8:**
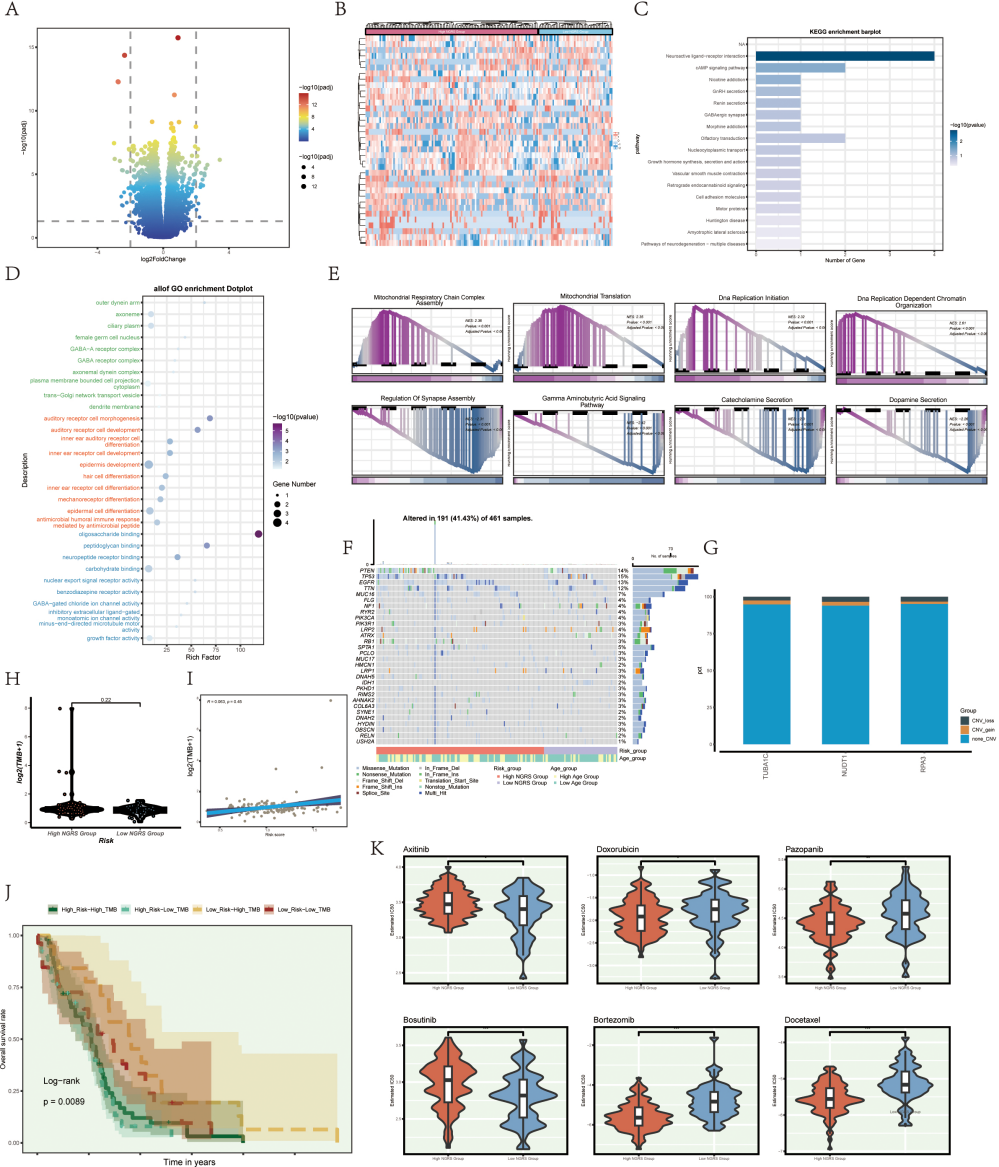
illustrates enrichment analysis, mutation analysis, and drug sensitivity analysis among different groups. **(A, B)** Volcano and heatmap illustrating the expression of various genes in the high and low C2 NUSAP+ glioma groups. **(C)** Enrichment results of various pathways are displayed in the KEGG enrichment analysis of differentially expressed genes. **(D)** Bar graph illustrating the outcomes of all Gene Ontology (GO) enrichment analyses (GOBP, GOCC, GOMF). **(E)** Enrichment scores of genes in various pathways are presented through GSEA scoring of GO-BP-enriched entries of differentially expressed genes. **(F)** Mutation waterfall plot illustrating the variances in the top 30 most frequently mutated genes in the two groups of somatic cells. The top column indicates the mutation load for each sample, and the right column indicates the total percentage of mutations in that gene in those samples. **(G)** CNV status of the model gene. **(H)** Violin plots are used to display the difference in mutation load between the high and low groupings of C2 NUSAP+ gliomas. **(I)** Scatter plot illustrating the correlation analysis of mutation load with C2 NUSAP+ glioma score. **(J)** Scoring is based on tumor mutation load, divided into four groups: high-risk high mutation load group, high-risk low mutation load group, low-risk high mutation load group, and low-risk low mutation load group. Curves showing the results of survival analysis for the four groups are presented. **(K)** Box diagram showing drug sensitivity analysis.

To compare the high and low NUSAP+ Glioma score groups, we employed volcano plots and heat maps to illustrate the expression patterns of distinct genes in each group (**Figures 8A, B**). To investigate the role of the C2 NUSAP+ Glioma subgroup in glioma pathogenesis, we conducted functional enrichment analysis on genes that distinguish these groups. Bubble plots presented the results of Gene Ontology (GO) enrichment analysis, highlighting that these genes are primarily involved in oligosaccharide binding, peptidoglycan binding, and pathways associated with auditory receptor cell development (**Figure 8D**). KEGG enrichment analysis, depicted in bar graphs, revealed significant associations with pathways such as neuroactive ligand-receptor interaction and the cAMP signaling pathway (**Figure 8C**). Additionally, Gene Set Enrichment Analysis (GSEA) scores indicated gene enrichment across various pathways, as shown in GO-BP-enriched entries for differentially expressed genes (**Figure 8E**).

### 3.8 Mutation analysis

We analyzed and visualized gene mutations within the tumor microenvironment (TME) to determine their correlation with immune components across two cell cohorts. This analysis revealed differences in the top 30 genes with the highest mutation frequencies within these mesenchymal cell cohorts. The vertical bar shows the mutation burden per sample, while the horizontal bar indicates the overall mutation prevalence of each gene across the samples (**Figure 8F**). No significant chromosomal copy number variations (CNVs) were detected in the genes analyzed, as shown by the lack of significant gains or losses in the CNV profile (**Figure 8G**). Violin plots were employed to examine mutation burden variations between high and low NUSAP+ Glioma score groups, revealing no statistically significant differences (**Figure 8H**). However, scatter plots showed a statistically significant correlation (P<0.05) between mutation load and NUSAP+ Glioma scores (**Figure 8I**). Tumors were classified into four categories based on mutation burden: high NUSAP+ Glioma score with high TMB, high NUSAP+ Glioma score with low TMB, low NUSAP+ Glioma score with high TMB, and low NUSAP+ Glioma score with low TMB. Survival analysis revealed that the group with a low NUSAP+ Glioma score and high TMB exhibited the most favorable survival outcomes, whereas the group with a high NUSAP+ Glioma score and low TMB showed the poorest prognosis (**Figure 8J**).

### 3.9 Drug sensitivity analysis

Violin plots were utilized to depict the differential responses to various medications between high and low NUSAP+ Glioma score groups, emphasizing variations in drug sensitivity (**Figure 8K**). The IC50 value for Axitinib was elevated in the high NUSAP+ Glioma score group relative to the low score group, indicating a decreased drug responsiveness in the former.

### 3.10 Silencing NUSAP inhibits proliferation, migration, and metastasis in glioma cells

**Figure 9:**
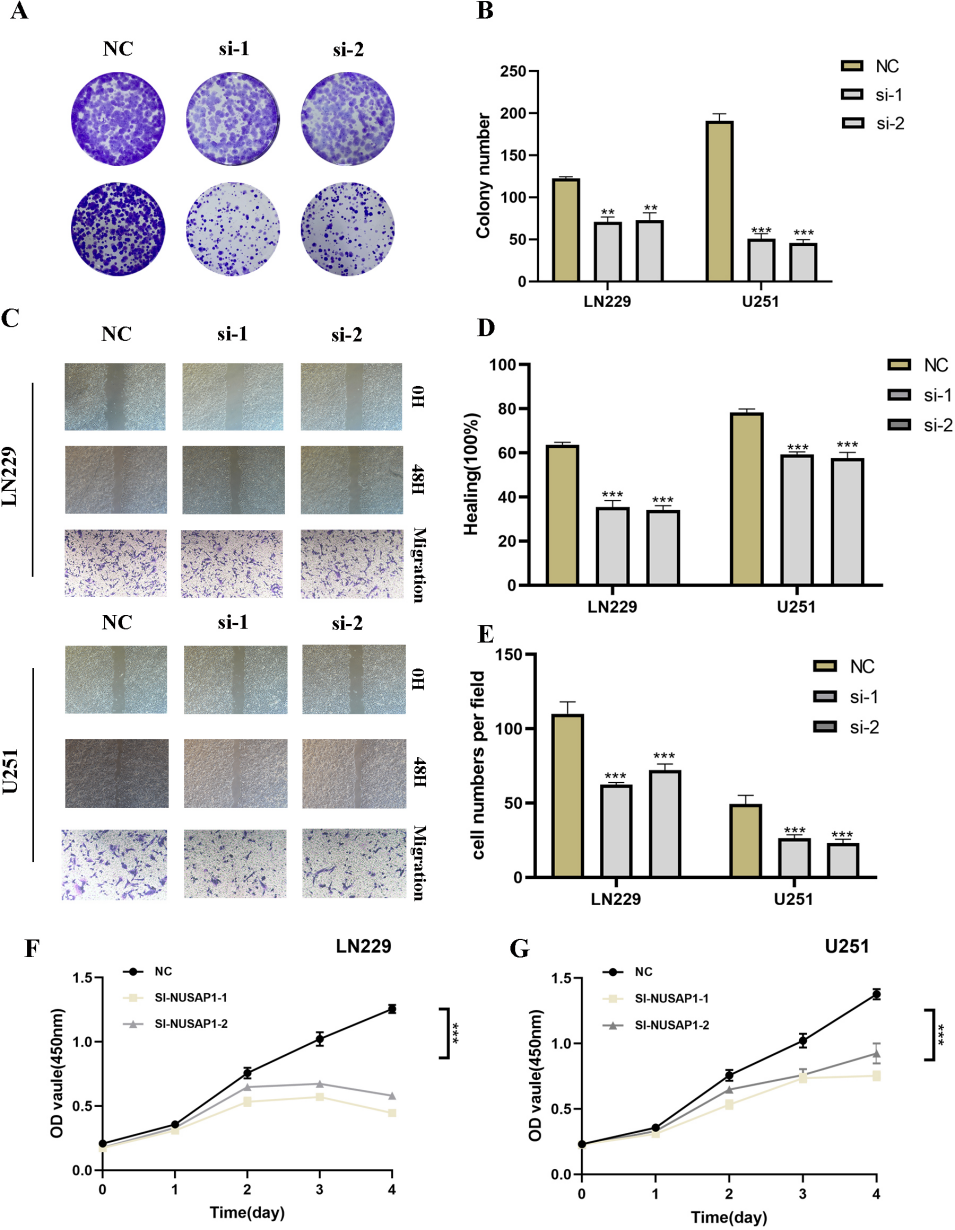
Silencing NUSAP1 Inhibits Proliferation, Migration and Metastasis while Promoting Apoptosis in Glioma Cells. **(A)** Colony formation assay was performed on U251 and LN229 glioma cells in the NC and si-NUSAP1 groups. Smaller colonies were observed in the si-NUSAP1 group, indicating that NUSAP1 silencing inhibits glioma cell proliferation. **(B)** Quantification of colony formation assay results showing a decrease in colony size in the si-NUSAP1 group compared to the NC group. **(C)** Transwell assay demonstrated a decrease in the migration ability of U251 and LN229 cells in the si-NUSAP1 group compared to the NC group. And scratch assay revealed a decrease in the migration ability of U251 and LN229 cells in the si-NUSAP1 group compared to the NC group. **(D)** Quantification of scratch assay results showing a decrease in wound closure percentage in the si-NUSAP1 group compared to the NC group. **(E)** Quantification of transwell assay results showing a decrease in the number of invading cells in the si-NUSAP1 group compared to the NC group. **(F)** CCK-8 assay further confirmed the inhibitory effect of NUSAP1 silencing on LN229 cells proliferation. **(G)** CCK-8 assay further confirmed the inhibitory effect of NUSAP1 silencing on U251 cells proliferation. **p<0.01, ***p<0.001.

In our investigation of NUSAP1’s influence on glioma, we conducted NUSAP1 gene knockdown via transfection, with confirmation of transfection efficiency using RT-qPCR (**Supplementary Figure 2**). Subsequently, colony formation assays were performed on U251 and LN229 glioma cell lines in both the negative control (NC) and si-NUSAP1 experimental groups (**Figure 9A**). The results indicated that inhibition of NUSAP1 led to reduced colony sizes in both U251 and LN229 cells, suggesting that downregulation of NUSAP1 impedes glioma cell proliferation (**Figure 9B**). To evaluate the effect of NUSAP1 on glioma cell migration, scratch and transwell assays were conducted, as depicted in **Figure 9C**. Our findings demonstrated a significant decrease in the migratory potential of U251 and LN229 cells following NUSAP1 knockdown (**Figures 9D, E**). Therefore, the suppression of NUSAP1 showed inhibitory effects on both glioma cell proliferation and migration, which were further validated using the CCK-8 assay (**Figures 9F, G**).

## 4 Discussion

Glioma continues to be one of the most difficult cancers to manage, as current treatment approaches have limited efficacy due to its aggressive behavior and intricate biological characteristics (30–32). Despite advancements in oncology, the progress of personalized medicine in glioma has been relatively slow, largely due to the tumor’s heterogeneity and the intricate interplay of genetic mutations (24, 33). However, the advent of single-cell technologies has the potential to revolutionize personalized treatment for glioma. By enabling detailed analysis of individual tumor cells, these technologies can uncover unique cellular subpopulations and molecular pathways driving tumor growth and resistance to therapy (34, 35). This detailed analysis not only deepens our comprehension of glioma biology but also aids in the creation of more targeted and effective personalized therapies, potentially enhancing patient outcomes (36, 37).

In our research, single-nucleus RNA sequencing (snRNA-seq) was conducted from nine glioma patients, uncovering the primary cell types and their intricate heterogeneity throughout tumor progression (38). This investigation provided an in-depth understanding of distinct cellular subpopulations and highlighted the impact of IDH1 mutation status on the distribution of these subpopulations and disease progression. Notably, we identified three major glioma cell subpopulations (C0 MALAT1+, C1 AKAP9+, and C2 NUSAP1+), which were highly correlated with tumor malignancy. Through trajectory analysis and interaction network studies, we uncovered the pivotal roles of these subpopulations in glioma progression and therapeutic response (39, 40).

These findings offer critical molecular insights for the development of personalized treatment strategies in glioma. For example, integrating specific cellular subpopulations identified in patient tumors with clinical prognosis could enhance the accuracy of predicting therapeutic responses, thereby supporting the formulation of individualized treatment plans (41). Moreover, the identification of intercellular signaling pathways, such as PTN and NCAM, and the specific gene expression patterns of NUSAP1 and AKAP9, accelerates the discovery of therapeutic targets and the development of novel drugs. Consequently, our study not only charts new directions for personalized glioma treatment but also presents an innovative approach to managing this highly heterogeneous tumor (42).

In parallel, our research has shown significant potential in advancing personalized treatment strategies for glioma, particularly in the context of genetic interventions aimed at modulating tumor progression. While NUSAP1 is recognized as an oncogene in several cancers, its role in glioma remains inadequately understood (43). Through NUSAP1 knockdown experiments, we elucidated its pivotal role in regulating glioma cell proliferation, migration, and distant metastasis. Both *in vitro* and *in vivo* assays—such as colony formation, scratch wound healing, transwell migration, and flow cytometry—demonstrated that NUSAP1 silencing significantly suppressed malignant behaviors of glioma cells and induced a marked increase in apoptosis. These results further underscore NUSAP1’s potential as a therapeutic target and lay the foundation for future precision medicine approaches (36).

Prior research has established a correlation between elevated NUSAP1 expression and adverse outcomes in various cancers, including breast, lung, and prostate. Our findings extend this knowledge to glioma, revealing similar mechanisms by which NUSAP1 contributes to tumorigenesis. Given the genetic and microenvironmental similarities between glioma and glioma, these insights support the potential of targeting NUSAP1 in glioma treatment (44, 45). By suppressing NUSAP1 expression, more precise tumor control may be achieved in the context of personalized therapy, offering better outcomes for patients (46).

Furthermore, with the rapid development of single-cell technologies, researchers can now capture tumor heterogeneity more accurately and identify key genes associated with tumor progression. NUSAP1, as one of these genes, has demonstrated its broad role in malignancies. This single-cell-based approach not only improves our understanding of tumor evolution but also guides the design of personalized treatment regimens, providing more precise and effective therapeutic options for patients across various cancer types (47, 48).

In conclusion, our findings provide new insights into the potential application of NUSAP1 in personalized glioma treatment. Future research exploring the interactions between NUSAP1 and other tumor-related genes may help develop more effective therapeutic strategies, advancing the progress of personalized medicine.

## 5 Conclusions

In conclusion, a thorough prognostic categorization and immune evaluation of glioma patients can be effectively executed through NUSAP1-linked methodologies. Furthermore, elevated NUSAP1 levels indicate a diminished overall survival (OS) expectation among glioma patients. These discoveries harbor promising implications for enhancing glioma detection, therapeutic strategies, and mechanistic investigations.

## Data Availability

The original contributions presented in the study are included in the article/**Supplementary Material**. Further inquiries can be directed to the corresponding authors.

## Supplementary material

Supplementary Figure 1 NUSAP1 gene transfection knock-down low efficiency verification. Compared with untransfected cells, the mRNA level of NUSAP1 gene was significantly decreased in the transfected knockdown group.

Supplementary Figure 2 The classification of GBM cells. According to the infer CNV results, we defined cells with high CNV levels as GBM cells. Note: C2 NUSAP+ gliomas show variations in drug sensitivities between high and low groups, as illustrated by violin plots. *p ≤ 0.05; **p ≤ 0.01; ***p ≤ 0.001 indicate a significant difference, while “ns” indicates a non-significant difference. N: Differences in drug sensitivities between high and low C2 NUSAP+glioma score groups are illustrated through violin plots. *, p ≤ 0.05; **p ≤ 0.01; ***p≤ 0.001 indicate a significant difference, and “ns” indicates a non-significant difference.
