# Supplementary figures and images for: Single-cell sequencing elucidates the mechanism of NUSAP1 in glioma and its diagnostic and prognostic significance
Source: Front Immunol. 2025 Feb 5;16:1512867. doi: 10.3389/fimmu.2025.1512867 (PMC11835852; doi:10.3389/fimmu.2025.1512867)

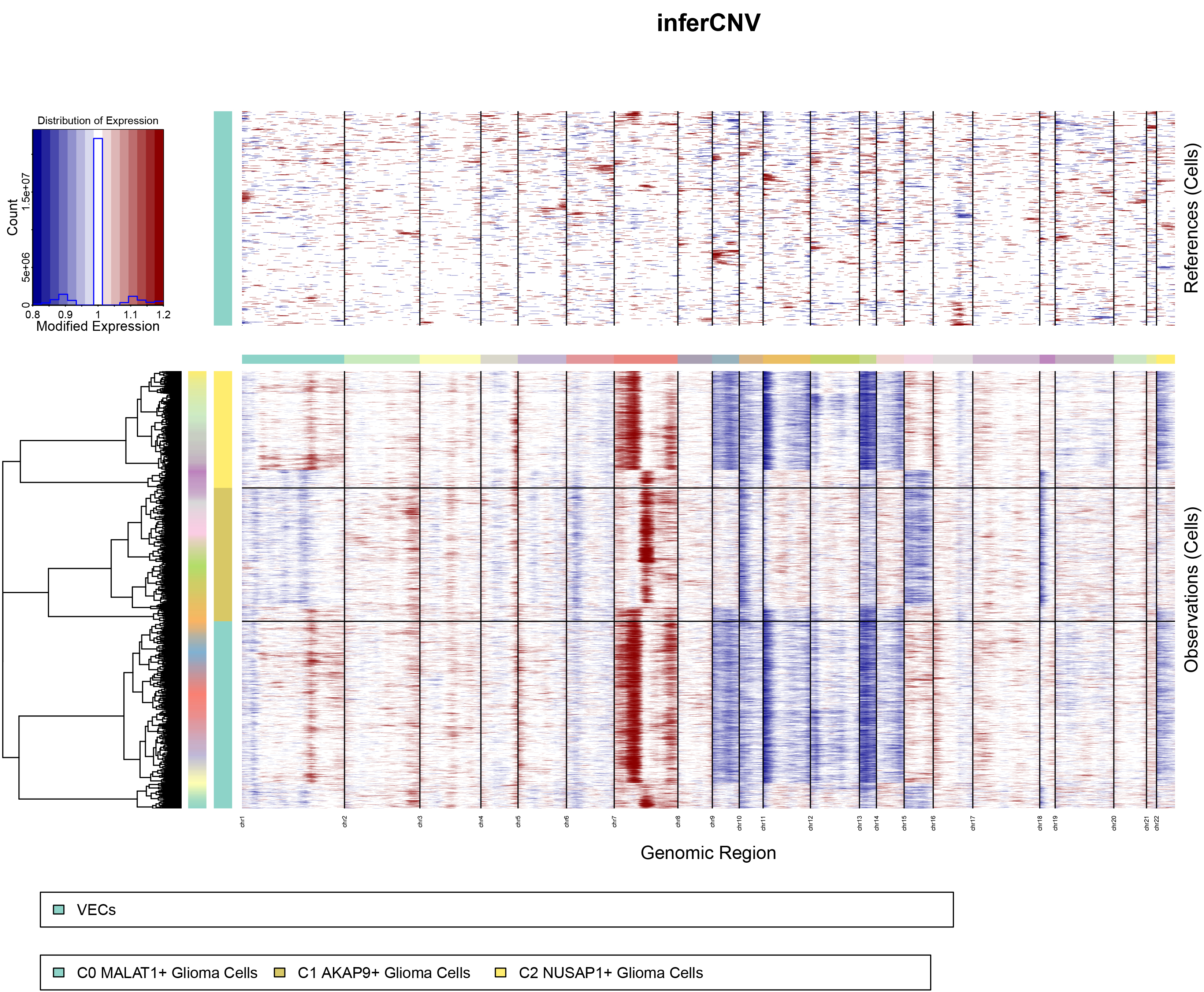

Supplement: Supplementary Figure 1 — NUSAP1 gene transfection knock-down low efficiency verification. Compared with untransfected cells, the mRNA level of NUSAP1 gene was significantly decreased in the transfected knockdown group. [file Image1.jpeg]

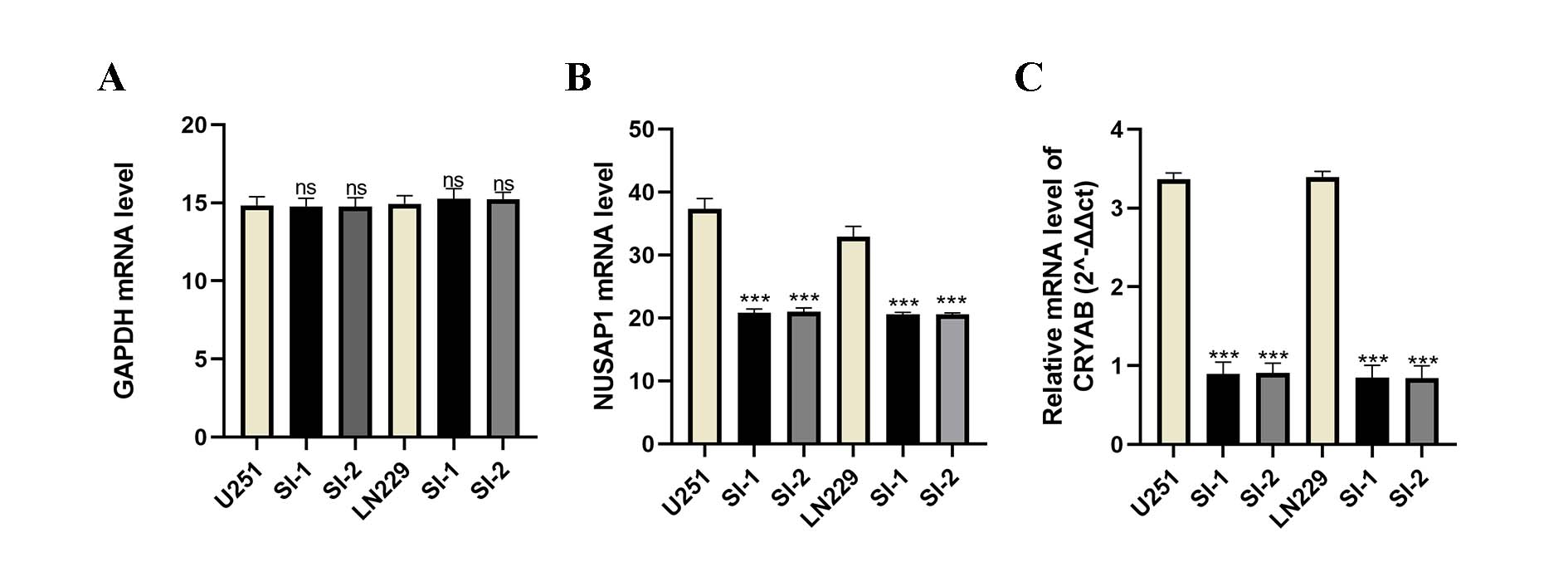

Supplement: Supplementary Figure 2 — The classification of GBM cells. According to the infer CNV results, we defined cells with high CNV levels as GBM cells. Note: C2 NUSAP+ gliomas show variations in drug sensitivities between high and low groups, as illustrated by violin plots. *p ≤ 0.05; **p ≤ 0.01; ***p ≤ 0.001 indicate a significant difference, while “ns” indicates a non-significant difference. N: Differences in drug sensitivities between high and low C2 NUSAP+glioma score groups are illustrated through violin plots. *, p ≤ 0.05; **p ≤ 0.01; ***p≤ 0.001 indicate a significant difference, and “ns” indicates a non-significant difference. [file Image2.jpeg]
