# Supplementary material for: Single-cell sequencing elucidates the mechanism of NUSAP1 in glioma and its diagnostic and prognostic significance
Source: Front Immunol. 2025 Feb 5;16:1512867. doi: 10.3389/fimmu.2025.1512867 (PMC11835852; doi:10.3389/fimmu.2025.1512867)
Supplement: Supplementary file 3 [file Table1.docx]

| **Oligonucleotides** | **Nucleotide sequence (5'-3')** |
| --- | --- |
| **siRNA** |  |
| Si-NUSAP1-1 | CCTCAGGTAACAGAGATTCAA |
| Si-NUSAP1-2 | GAGCACCAAGAAGCTGAGAAT |
|  |  |
| **Primer** |  |
| GAPDH | GGCCTCCAAGGAGTAAGACC (forward) |
|  | AGGGGAGATTCAGTGTGGTG (reverse) |
| NUSAP1 | ACCAGACGAGCACCAAGAAG (forward) |
|  | TGATGGGCTGCTTCAGTTCA (reverse) |
|  |  |

**Table S1. Oligonucleotides used in research**
